# Critical appraisal of ^131^I SPECT/CT in differentiated thyroid cancer: Implications for risk stratification and personalized management in thyroidology

**DOI:** 10.1016/j.clinsp.2026.100964

**Published:** 2026-05-11

**Authors:** Ilker Sengul, Demet Sengul

**Affiliations:** a Thyroidology Unit, Giresun University Faculty of Medicine, Turkey; b Division of Endocrine Surgery, Giresun University Faculty of Medicine, Turkey; c Department of General Surgery, Giresun University Faculty of Medicine, Turkey; d Department of Pathology, Giresun University Faculty of Medicine, Turkey

*Dear Editor,*

The management, prognosis, and follow-up of Differentiated Thyroid Carcinoma (DTC) remain critical and controversial areas in thyroidology [1, 2, 3] We discuss the article by Heinrich and colleagues regarding the prognostic implications of Lymph Node Metastases (LNM) detected on post-therapy ^131^I SPECT/CT. Heinrich et al. [4] present a significant single-center cohort analysis, providing valuable insights into how LNM characteristics (size and ^131^I avidity) impact complete response and progression-free survival. Their conclusion that SPECT/CT offers critical information for tailored treatment is highly relevant. The findings provide valuable insights, particularly regarding the impact of LNM characteristics (size and avidity) on Complete Response (CR) and Progression-Free Survival (PFS), and the effectiveness of subsequent treatment modalities, such as redo surgery versus second Radioiodine Therapy (RAI). The conclusion that SPECT/CT provides critical information for patient outcomes and tailored treatment decisions is highly relevant for clinical practice. However, we wish to offer some constructive and critical remarks regarding certain aspects and limitations acknowledged within the study itself, which could enhance the interpretation of its findings and inform future research.

Firstly, Several limitations warrant attention: i) The small sample size in the CT1/S0 subgroup precludes definitive conclusions regarding redo surgery. ii) The exclusion of Thyroglobulin (Tg) levels limits the holistic understanding of outcomes, as Tg is an independent risk factor for recurrence. iii) The retrospective design may introduce biases in treatment outcome analysis. iv) The lack of standardized, quantitative metrics and interobserver comparison in image evaluation remains a significant constraint. The authors themselves note that for these -negative lesions, RAI is presumed ineffective, making reoperation the only effective treatment option. The intriguing observation that this subgroup did not exhibit late progression, unlike enlarged-positive LNM, is acknowledged to be a statistical effect of the small sample size, raising questions about the true nature and aggressiveness of these LNM. Consequently, further investigation with larger cohorts would be beneficial to clarify these points. Secondly, the exclusion of biochemical markers, particularly Thyroglobulin (Tg) levels, from the analysis may warrant discussion. The authors explicitly state that this was performed in order to evaluate imaging modalities in isolation and avoid confounding influences from biochemical markers. While this focused approach is understandable, it also limits a comprehensive understanding of patient outcomes, as Tg levels are a well-established early marker for recurrence and an independent risk factor for persistent/recurrent disease and CR. As the study itself suggests, future research incorporating both imaging findings and biochemical markers would likely provide a more holistic prognostic model. Thirdly, the study's retrospective, monocentric design, while ensuring homogeneity in laboratory assays and surgical protocols, inherently introduces limitations. The initial treatments and subsequent diagnostic decisions during follow-up were not based on a uniform prospective protocol, and other risk factors beyond LNM characteristics that might have influenced treatment decisions (reoperation vs. RAI) were not considered. This could introduce biases into treatment outcome analysis. Finally, some methodological points related to image evaluation deserve mention: i) The visual definition of pathological uptake, particularly using salivary gland uptake as a reference, is noted as an approach not previously used in DTC and lacks objective measurements. This absence of a standardized, quantitative metric is a significant limitation, ii) The analysis of all post-therapy SPECT/CT images by a single experienced nuclear medicine specialist without an interobserver comparison could introduce subjective bias, despite the interpreter's expertise. Inter-rater reliability assessment is a standard practice that would have strengthened the findings, iii) The absence of contrast medium in the CT scans meant that contrast-based pathological characteristics, such as enhancement patterns, could not be assessed, thereby limiting the diagnostic power of the CT imaging, and iv) The study focused specifically on the size and avidity of LNM, while not considering the number of pathological lymph nodes, which other studies have included as a prognostic factor. The omission of this established prognostic factor warrants further consideration. Future prospective multicenter studies should integrate imaging findings with biochemical markers to provide a more holistic prognostic model. Standardizing quantitative metrics for ^131^I uptake will be essential for reproducibility.

Despite these points, the study by Heinrich and colleagues [4] makes a significant contribution to our understanding of the prognostic value of SPECT/CT in DTC. The detailed characterization of LNM based on size and avidity, along with its implications for treatment strategy, provides crucial evidence for informed clinical decision-making. We anticipate that addressing these limitations in future prospective, multicenter studies, potentially integrating biochemical markers and standardized quantitative imaging assessments, will further refine risk stratification and personalize management for DTC patients. Clinicians should interpret SPECT/CT findings in conjunction with biochemical data. We invite multicenter, prospective trials that combine SPECT/CT with Tg monitoring and contrast-enhanced CT to validate a unified prognostic model for DTC. In essence, we thank Heinrich et al. [4] for their valuable study on SPECT/CT in DTC for the thyroidology community [1, 2, 3, 4, 5, 6, 7] As thyroidology moves toward personalized management, integrating functional imaging with biochemical and histopathological data will be essential. This correspondence calls for methodological rigor in future prognostic studies to ensure that SPECT/CT findings translate into clinically actionable insights.

ARTICLE INFO

*Article history*:

Received 15 November 2025

Accepted 23 January 2026

Keywords: Thyroid Gland, Differentiated Thyroid Carcinoma, 131I SPECT/CT, Nuclear Medicine, Thyroidology, Thyroidologists, Risk Stratification, Personalized Medicine, Lymph Node Metastasis, Thyroglobulin, Prognostic Imaging

## Ethics approval

Not applicable.

## Consent to participate

Not applicable.

## Consent to publish

Not applicable.

## Code availability

Not applicable.

## Authors’ contributions

Ilker Sengul: Conceptualization; formal analysis; ınvestigation; methodology; project administration; resources; validation; visualization; writing-original draft; writing-review & editing.

Demet Sengul: Conceptualization; formal analysis; ınvestigation; methodology; software, supervision; validation; visualization; writing-original draft; writing-review & editing.

## Funding

None declared.

## Data availability statement

The datasets generated and/or analyzed during the current study are available from the corresponding author upon reasonable request.

## Declaration of competing interest

The authors declare no conflicts of interest.
